# Endometrial adenocarcinoma arising from adenomyosis: A case report and literature review

**DOI:** 10.1097/MD.0000000000045810

**Published:** 2026-01-30

**Authors:** Xiaoxuan Liu, Yang Yue, Guoyun Wang

**Affiliations:** a Department of Obstetrics and Gynecology, Shandong Provincial Hospital, Shandong University, Jinan, Shandong, China; b Medical Integration and Practice Center, Cheeloo College of Medicine, Shandong University, Jinan, Shandong, China; c Jinan Key Laboratory of Diagnosis and Treatment of Major Gynaecological Disease, Jinan, Shandong Province, China; d Gynecology Laboratory, Shandong Provincial Hospital, Jinan, Shandong Province, China; e Gynecology Laboratory, Medical Science and Technology Innovation Center, Shandong First Medical University & Shandong Academy of Medical Sciences, Jinan, Shandong Province, China; f Department of Gynecology and Obstetrics, Shandong Provincial Hospital Affiliated to Shandong First Medical University, Jinan, Shandong Province, China; g Shandong Key Laboratory of Reproductive Research and Birth Defect Prevention, Shandong First Medical University, Jinan, Shandong Province, China; h Shandong Provincial Health Commission Key Laboratory of Prevention and Treatment for Major Gynecological Diseases, Shandong Provincial Hospital Affiliated to Shandong First Medical University, Jinan, Shandong Province, China.

**Keywords:** adenomyosis, case report, endometrioid adenocarcinoma, immunohistochemistry, malignant transformation, pathology

## Abstract

**Background:**

Endometrial carcinoma arising from adenomyosis (EC-AIA) is remarkably uncommon, and its underlying molecular mechanisms are not yet fully elucidated. This knowledge gap is particularly significant given that most reported EC-AIA cases are well-differentiated and hormone receptor-positive, creating a critical need to characterize the rare, aggressive variants and their clinical implications.

**Objective:**

This study aims to address this gap by presenting a unique case of poorly differentiated endometrioid adenocarcinoma with adenomyosis, exploring their potential association, and to synthesize current understanding through literature review to inform clinical decision-making.

**Case presentation:**

A 45-year-old woman with a history of adenomyotic lesion resection presented with abnormal uterine bleeding. Postoperative pathology confirmed poorly differentiated endometrioid adenocarcinoma (International Federation of Gynecology and Obstetrics IIIC2 stage), with immunohistochemistry showing estrogen receptor/progesterone receptor (PR) negativity, p53 mutation pattern, and nonspecific molecular profile. Concurrent adenomyosis (0.6 cm) was identified, though direct histological transition between adenomyosis and carcinoma was not established. The patient underwent cytoreductive surgery and platinum-based chemotherapy.

**Discussion:**

Our analysis reveals that the relationship between adenomyosis and endometrial carcinoma remains debated. This case (characterized by high-grade histology, hormone receptor negativity, and widespread metastases) provides crucial evidence diverging from the classic EC-AIA profile (typically well-differentiated and hormone-sensitive), implying a distinct malignant transformation mechanism. These findings challenge the conventional understanding of EC-AIA and highlight the spectrum of its clinical presentations.

**Conclusion:**

This study underscores that the management of suspected malignant transformation of adenomyosis requires multidisciplinary evaluation. More importantly, our findings demonstrate that aggressive treatment should be initiated even without definitive pathological confirmation when clinical suspicion is high. The significance of this work lies in its contribution to recognizing the heterogeneous nature of EC-AIA, urging future research to focus on elucidating molecular mechanisms and developing personalized therapeutic strategies for these aggressive variants.

## 1. Introduction

Endometrial carcinoma represents one of the most common malignancies of the female reproductive system. While most cases arise de novo, rare instances may originate from malignant transformation of adenomyosis. Adenomyosis, a benign condition characterized by the invasion of endometrial glands and stroma into the myometrium, has an unclear carcinogenic mechanism with only sporadic cases reported in literature. Current evidence indicates that most adenomyosis-associated malignant transformations present as well-differentiated, hormone receptor-positive endometrioid adenocarcinoma, while poorly differentiated variants are exceptionally uncommon.

Previous studies suggest that adenomyosis may coexist with endometrial carcinoma in 18% to 66% of cases, although carcinoma arising directly within adenomyotic foci (endometrial carcinoma arising in adenomyosis [EC-AIA]) is exceedingly rare, accounting for approximately 1% of cases.^[1]^ Epidemiological evidence indicates that women with adenomyosis or endometriosis carry a significantly higher risk of developing endometrial carcinoma compared to the general population (odds ratio ≈ 3.6).^[2]^

The molecular mechanisms underlying this transformation remain incompletely understood. Potential contributors include chronic oxidative stress,^[3]^ PTEN/PIK3CA mutations,^[4]^ alterations in the hormonal microenvironment,^[5]^ epigenetic dysregulation,^[6]^ and impaired immune surveillance. Clinically, most reported EC-AIA cases present as well-differentiated, hormone receptor-positive endometrioid adenocarcinoma, while poorly differentiated and hormone receptor-negative variants are exceptionally uncommon.

This case report describes a 45-year-old woman with prior surgical resection of adenomyotic lesions who presented with abnormal uterine bleeding and was subsequently diagnosed with poorly differentiated endometrioid adenocarcinoma (International Federation of Gynecology and Obstetrics [FIGO] IIIC2 stage) with extensive metastases. Although direct histopathological evidence of malignant transformation was not established, the patient’s medical history, imaging findings, and immunohistochemical profile collectively suggest adenomyosis as a potential origin. The study objectives were to: characterize the clinicopathological features of adenomyosis-associated endometrial carcinoma; evaluate diagnostic criteria and therapeutic approaches; and establish a reference framework for managing similar cases.

Current understanding of the molecular mechanisms underlying this malignant transformation remains limited. Future investigations incorporating genomic profiling and larger clinical cohorts are imperative to elucidate its biological behavior and prognostic determinants.

## 2. Case presentation

A 45-year-old Chinese woman (divorced, nulliparous) presented with a 4-month history of persistent metrorrhagia without obvious precipitating factors. Her medical history included severe dysmenorrhea requiring regular analgesic use. Two months prior, an outside hospital pelvic ultrasound had revealed uterine fibroids, for which she received traditional Chinese medicine treatment. Subsequent evaluation 1 month before admission showed human papillomavirus testing negative, ThinPrep cytologic test demonstrating atypical glandular cells suspicious for neoplasia, and ultrasound revealing endometrial thickening (1.2 cm).

At our institution, diagnostic curettage pathology confirmed adenocarcinoma in both cervical and endometrial specimens, suggestive of high-grade endometrioid adenocarcinoma. The patient had a history of multiple ovarian cystectomies and adenomyotic lesion resection.

Imaging findings at our hospital included pelvic ultrasound showing an enlarged uterus (8.5 × 6.9 × 6.6 cm) with heterogeneous endometrium (1.4 cm defect), poor vascularity, and intracavitary mass; anterior wall fibroid (3.5 × 3.1 cm); and dilated left fallopian tube (4.9 × 2.7 cm) with solid components. Pelvic magnetic resonance imaging further demonstrated endometrial thickening with restricted diffusion on restricted diffusion on diffusion-weighted imaging, right upper uterine mass (7.1 × 6.1 × 6.2 cm) with hemorrhagic features, vaginal metastasis (1.2 × 1.1 cm) at mid-upper segment, left tubal nodule (0.8 cm) and pelvic lymphadenopathy (0.7 cm) showing diffusion-weighted imaging restriction. The comprehensive imaging findings were consistent with FIGO stage III endometrial carcinoma with tubal/vaginal metastases and suspected pelvic nodal involvement, concurrent with uterine fibroids. Tumor markers showed cancer antigen 125 at 69.30 U/mL, human epididymis protein 4 at 297.00 pmol/L, and risk of ovarian malignancy algorithm index of 86.01% (premenopausal) and 71.79% (postmenopausal).

**Figure 1.**
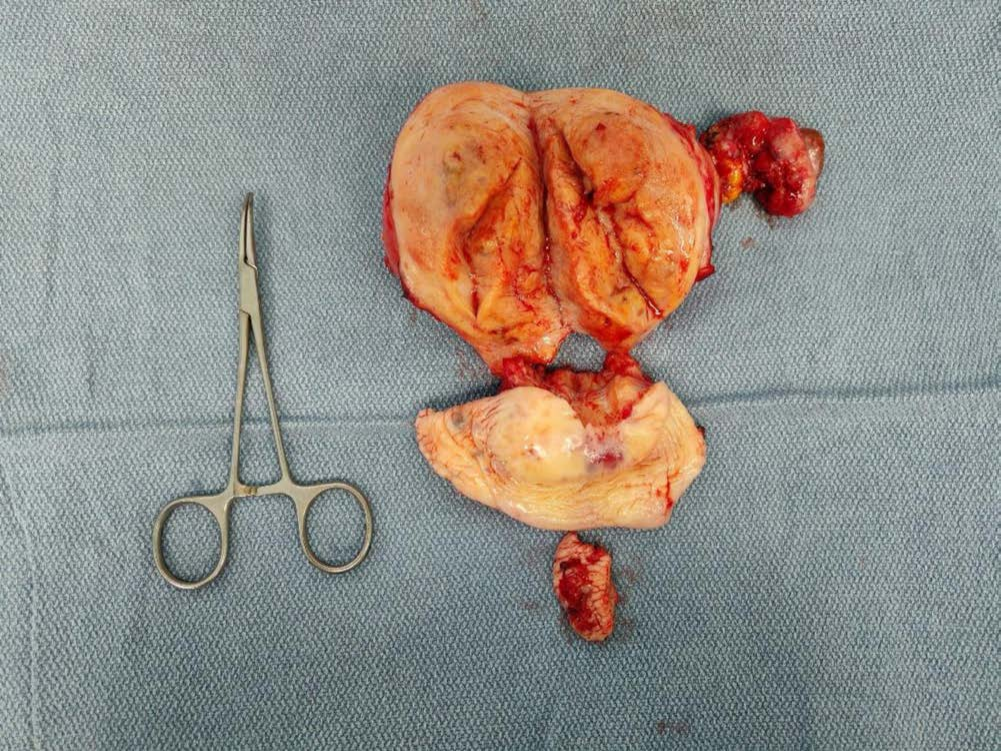
Gross pathology. The uterine myometrium appears firm and coarse, with a metastatic lesion visible on the posterior vaginal wall. The right adnexa is absent.

The patient underwent radical abdominal hysterectomy with left salpingo-oophorectomy, pelvic/para-aortic lymphadenectomy, omentectomy, rectal/vaginal metastatic lesion resection, and adhesiolysis. Intraoperative findings included 50 mL serosanguinous ascites, uterine enlargement (equivalent to 12-week gestation) with diffuse adhesions, multiple metastatic deposits in rectouterine pouch (largest 0.8 × 0.6 cm), omental miliary lesions, and 2 cm vaginal vault metastasis. The right adnexa was absent while left ovarian appearance was normal (Fig. 1).

**Figure 2.**
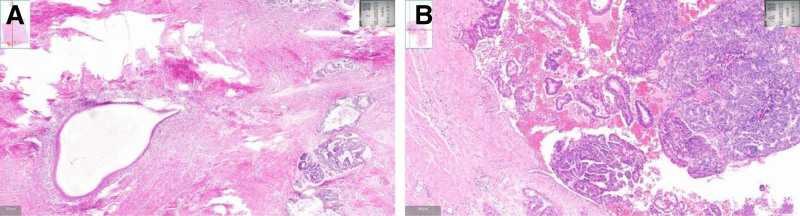
Pathological examination. (A) Coexistence of adenomyosis and endometrial carcinoma is observed; (B) normal endometrial glandular tissue, inflammatory hyperplasia, and endometrial carcinoma are present together.

**Figure 3.**
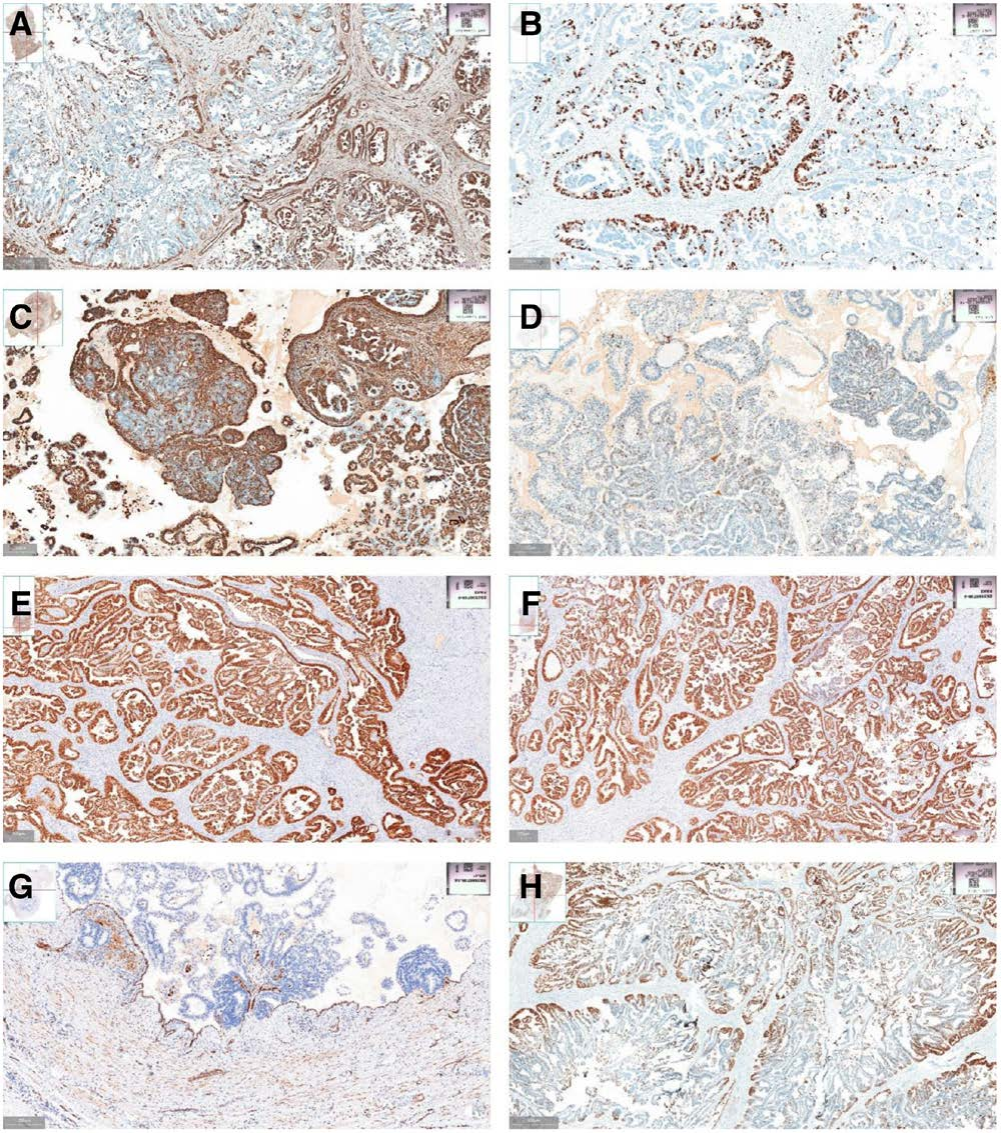
Immunohistochemistry. (A) Vimentin; (B) Ki67; (C) vimentin; (D) p53; (E) PAX2; (F) PAX8; (G) WT1; (H) TTF1. Results: WT1 (−), vimentin (+), p53 (+), Ki-67 (10%), PAX2 (+), TTF1 (+), PAX8 (+).

Postoperative pathology revealed poorly differentiated carcinoma (5.2 × 3.8 cm), morphologically and immunohistochemically consistent with endometrioid adenocarcinoma, with myometrial invasion > 2/3 depth, cervical stromal involvement, and absent lymphovascular space invasion. Additional findings included focal adenomyosis (0.6 cm) and 3 uterine leiomyomas (1–3.2 cm) (Fig. 2). Metastases were confirmed in left adnexa, omentum, bowel, vaginal wall, and lymph nodes (left pelvic 5/9, right pelvic 4/5, right common iliac 3/6, para-aortic 1/1). Immunohistochemistry showed positive staining for vimentin, p16 (focal), p53, PAX8, TTF1, and negative for estrogen receptor (ER), progesterone receptor (PR), WT-1, GATA-3, NapsinA, with Ki-67 index of 10% to 30% (Fig. 3). Molecular profiling demonstrated nonspecific molecular profile. The patient received adjuvant chemotherapy with paclitaxel/carboplatin and tolerated the regimen well.

## 3. Outcome and follow-up

The patient’s postoperative course was uneventful, and she was discharged on the seventh day after surgery. She subsequently received 6 cycles of adjuvant chemotherapy with paclitaxel (175 mg/m²) and carboplatin (AUC 5). Follow-up imaging at 3 months post-chemotherapy showed no evidence of disease recurrence, and her serum CA-125 level had normalized to 15.2 U/mL. The patient remains under close surveillance with quarterly clinical, radiological, and tumor marker assessments.

## 4. Discussion

### 4.1. Association between adenomyosis and endometrial carcinoma

Adenomyosis is a common benign condition characterized by the invasion of endometrial glands and stroma into the myometrium. The clinical correlation between adenomyosis and endometrial carcinoma (EC) has attracted significant attention. Current studies indicate that while the coexistence of these 2 conditions is relatively common (observed in 18–66% of EC patients), endometrial carcinoma arising directly from adenomyotic lesions is exceedingly rare, accounting for only approximately 1% of cases.^[7,8]^ Notably, patients with adenomyosis/endometriosis demonstrate a significantly higher incidence of endometrial carcinoma compared to the general population (odds ratio = 3.6),^[2]^ suggesting a potential pathophysiological link between these conditions.

In 1959, Colman and Rosenthal modified Sampson criteria to apply to carcinomas developing from adenomyosis: carcinoma should be absent from the normally situated endometrium and anywhere else in the pelvis; the carcinoma should be actually observed to be arising from the epithelium of the areas of adenomyosis and not invading from another source; and endometrial stromal cells should surround the aberrant glands to support a diagnosis of adenomyosis.^[9]^ The patient had a history of adenomyotic lesion resection in 2019. The current postoperative pathology showed poorly differentiated endometrioid adenocarcinoma (hormone receptor-negative, p53 mutant-type, Ki-67 30%) with extensive metastases, consistent with high-grade carcinoma characteristics. The pathological report clearly identified adenomyosis (measuring 0.6 cm microscopically). Combined with the patient’s deep myometrial invasion (involving two-thirds of the uterine wall) and multifocal growth pattern (metastases to omentum, bowel, and vaginal wall), these findings suggest adenomyosis may have served as a potential origin for malignant transformation. Currently, the diagnosis of malignant transformation of adenomyosis requires meeting 2 key criteria: pathological evidence of a transitional zone between adenomyosis and cancerous tissue; and molecular confirmation of shared driver gene mutations (such as PTEN and PIK3CA). This case has diagnostic limitations due to the lack of molecular comparison of current lesions, and future similar cases are recommended to undergo whole-genome sequencing to clarify clonal origin. It is noteworthy that most reported cases of malignant transformation of adenomyosis in the literature are well-differentiated, ER/PR-positive tumors, while the poorly differentiated and hormone receptor-negative characteristics of this case may represent a more aggressive subtype, requiring further investigation into its molecular mechanisms.

### 4.2. Possible molecular mechanisms

The molecular mechanisms underlying malignant transformation of adenomyosis remain incompletely understood, with current evidence suggesting a multifactorial pathogenesis. Chronic oxidative stress serves as a key contributing factor,^[2,3]^ playing a significant role in the malignant transformation process similar to that observed in endometriosis-associated carcinogenesis, particularly notable in cases of cystic adenomyosis transforming into clear cell carcinoma.^[3]^ Concurrently, genetic abnormalities including PTEN and PIK3CA mutations, epigenetic dysregulation, and tumor suppressor gene inactivation collectively form the molecular foundation of malignant transformation.^[4]^ Additionally, alterations in hormonal microenvironment,^[8,10]^ dysregulation of the TSC2-mTOR autophagy pathway,^[6]^ and endometrial microbiota imbalance^[5]^ may also participate in this process. However, the interactive network among these factors and their precise regulatory mechanisms require further elucidation through future research.

### 4.3. Clinical management, preventive strategies, and future directions

Currently, there is no unified consensus regarding the treatment of malignant transformation of adenomyosis, with most evidence derived from small-scale studies or case reports.^[8,11,12]^ The present case was managed with cytoreductive surgery combined with platinum-based chemotherapy, consistent with standard treatment for advanced endometrial carcinoma. However, several distinctive features warrant attention: hormone receptor negativity (ER/PR−) indicates ineffectiveness of endocrine therapy, necessitating reliance on chemotherapy and targeted therapies; the p53 mutant phenotype may demonstrate sensitivity to immune checkpoint inhibitors; and extensive metastases (involving omentum, vagina, and lymph nodes) suggest poor prognosis with a 5-year survival rate below 30%. This case highlights that endometrial carcinoma patients with adenomyosis history should receive aggressive treatment as high-risk carcinoma (including intensive chemotherapy, targeted therapy, or immunotherapy), along with close surveillance for recurrence, even when malignant transformation cannot be definitively confirmed.

In addition to treatment, preventive and follow-up strategies are essential. Women with adenomyosis, particularly those with early-onset disease or prolonged estrogen exposure, may benefit from closer gynecologic surveillance. Unexplained abnormal uterine bleeding should prompt timely imaging and histological assessment. Molecular profiling may identify high-risk subgroups and guide early interventions. As suggested by recent literature,^[13]^ systematic follow-up protocols may reduce diagnostic delays.

Regarding prognosis, most studies support a protective effect of adenomyosis on endometrial carcinoma. Patients with coexisting adenomyosis typically present with more favorable tumor characteristics, including endometrioid subtype, early FIGO stage (I–II), low-grade histology, absence of deep myometrial invasion or lymphovascular space invasion, and significantly prolonged overall survival (HR = 0.51).^[14–16]^ However, this protective effect may result from clinical selection bias (such as younger patient age and less aggressive tumor biology) rather than independent molecular mechanisms, as key molecular markers (e.g., mismatch repair deficiency and p53 mutations) show no significant differences between groups.^[17]^ In contrast, EC-AIA often demonstrates more aggressive biological behavior and poorer prognosis,^[1,2,7]^ due to its intramyometrial location, diagnostic challenges (approximately 20% are confirmed postoperatively), and frequent advanced stage at diagnosis.

The relationship between adenomyosis and EC exhibits dual characteristics: their frequent coexistence suggests shared risk factors (e.g., epithelial–mesenchymal transition, KRAS mutations),^[18,19]^ while adenomyosis may improve prognosis through selection of low-risk subgroups or microenvironment modulation. In contrast, EC-AIA requires management as a distinct entity. Current treatment for adenomyosis malignant transformation primarily involves surgical intervention. Future directions should incorporate large-scale prospective studies and multi-omics analyses to elucidate causal relationships and identify potential therapeutic targets,^[4,15]^ thereby advancing personalized treatment strategies (including targeted therapy and optimized conservative surgery) and multidisciplinary collaboration (integrating molecular diagnostics and imaging modalities).^[10,20]^ Clinical practice should maintain heightened vigilance for high-risk patients (e.g., postmenopausal women with progressive lesions), with additional evidence to be accumulated through multicenter studies.^[3,8]^

### 4.4. Study limitations

This study has several limitations inherent to its design as a single case report. Firstly, the definitive diagnosis of EC-AIA requires both histological evidence of a transition zone between benign adenomyosis and carcinoma and molecular confirmation of clonality. In our case, while adenomyosis was present, a clear histological transition was not established, and molecular comparison between the adenomyotic focus and the carcinoma was not performed due to technical and resource constraints. Secondly, the findings from a single case cannot be generalized to the broader population. The unique characteristics of this case, such as its high-grade, hormone receptor-negative phenotype, may represent a rare subtype of EC-AIA. Future multicenter studies with integrated genomic profiling are needed to validate our observations.

## 5. Conclusion

Malignant transformation of adenomyosis into endometrial carcinoma represents an exceptionally rare clinical entity. This case of poorly differentiated, hormone receptor-negative, p53-mutated high-grade endometrioid adenocarcinoma suggests adenomyosis may serve as a potential high-risk factor, though its transformation mechanisms require further investigation. Future research should focus on: delineating molecular characteristics through multi-omics profiling; evaluating targeted therapies and immunotherapies for this unique subtype; and establishing multicenter collaborative networks to accumulate robust clinical evidence for standardized management. For endometrial carcinoma patients with coexisting adenomyosis, we recommend adherence to high-risk carcinoma treatment protocols even without definitive evidence of malignant transformation, to optimize patient outcomes.

## Acknowledgments

We sincerely thank the support of the colleagues of Shandong Provincial Hospital Affiliated to Shandong First Medical University.

## Author contributions

**Data curation:** Yang Yue.

**Writing – original draft:** Xiaoxuan Liu.

**Writing – review & editing:** Guoyun Wang.
